# Impact of OMICS Technologies in Our Understanding of the Pathogenesis of Peri‐Implantitis

**DOI:** 10.1002/cre2.70350

**Published:** 2026-04-13

**Authors:** Farah Asa'ad, Akira Hasuike, Koki Yoshida, Ronaldo Lira‐Junior, Akhilanand Chaurasia, Paula Milena Giraldo‐Osorno, Carlos Garaicoa‐Pazmino

**Affiliations:** ^1^ Department of Oral Biochemistry, Institute of Odontology, Sahlgrenska Academy University of Gothenburg Göteborg Sweden; ^2^ Department of Periodontology Nihon University School of Dentistry Tokyo Japan; ^3^ Division of Oral Medicine and Pathology, Department of Human Biology and Pathophysiology, School of Dentistry Health Sciences University of Hokkaido Hokkaido Japan; ^4^ Division of Oral Diagnostics and Surgery, Department of Dental Medicine Karolinska Institutet Stockholm Sweden; ^5^ Department of Oral Medicine and Radiology King George's Medical University Lucknow India; ^6^ Department of Medical and Translational Biology Umeå University Umeå Sweden; ^7^ Department of Periodontics University of Iowa College of Dentistry Iowa City Iowa USA; ^8^ Research Center, School of Dentistry, Universidad de Especialidades Espiritu Santo Samborondón Ecuador

**Keywords:** epigenomics, genomics, metabolomics, peri‐implantitis, proteomics, transcriptomics

## Abstract

**Objectives:**

To evaluate the contribution of OMICS technologies to the understanding of peri‐implantitis pathogenesis from a host immune perspective.

**Materials and Methods:**

A narrative review was conducted based on electronic searches of PubMed, MEDLINE, and Google Scholar up to October 2025, complemented by manual screening of reference lists. Search terms combined “peri‐implantitis” with OMICS‐related keywords, including genomics, epigenomics, transcriptomics, proteomics, metabolomics, RNA sequencing, single‐cell and spatial transcriptomics, multi‐omics, and machine learning. Studies were selected based on clinical relevance and their contribution to understanding peri‐implantitis pathogenesis from a host immune perspective.

**Results:**

Among the studies included, most focused on transcriptomic analyses, while fewer investigated genomics, epigenomics, proteomics, or metabolomics. Integration across OMICS layers highlights peri‐implantitis as a multilayered host–microbiome molecular ecosystem. Genomic variants affecting metal ion binding, cytoskeletal organization, and cell adhesion may predispose tissues to heightened immune sensitivity. Epigenomic analyses revealed differential DNA methylation of immune‐regulatory and signaling genes, linking environmental exposures, such as smoking, to altered host responses. Transcriptomic studies, including bulk, single‐cell, and spatial approaches, demonstrated dysregulated immune signaling, pro‐inflammatory fibroblast–neutrophil interactions, oxidative stress, and dysregulated tissue remodeling. Proteomic profiling of peri‐implant crevicular fluid confirmed elevated neutrophil‐derived antimicrobial proteins and inflammatory mediators, reflecting active host defense responses. Metabolomic studies identified disease‐specific alterations in amino acids, organic acids, and polyamines, which correlate with pathogenic microbial taxa and modulate immune and tissue responses. Collectively, these findings reveal convergent pathways of immune dysregulation, extracellular matrix disruption, tissue remodeling, and host–microbiome crosstalk as central features of peri‐implantitis.

**Conclusions:**

OMICS analyses show that peri‐implantitis is a complex host–microbiome molecular ecosystem. Integrated molecular insights provide a foundation for biomarker development, predictive diagnostics, and targeted interventions. However, future studies with larger cohorts and functional validation are needed to support clinical translation.

## 1 Introduction

Over the past few decades, dental implants have emerged as a widely accepted alternative to replace missing natural teeth (Chappuis et al. 2013). However, an increasing number of patients and clinicians face challenges due to biological and prosthetic complications following implant placement. Among these, peri‐implant diseases pose a considerable threat to implant success, compromising long‐term stability and function.

Peri‐implantitis is a biofilm‐associated pathological condition occurring in tissues around dental implants, characterized by inflammation in the peri‐implant mucosa and progressive loss of supporting bone (Berglundh et al. 2018). Reported prevalence rates vary widely across studies, ranging from 1% to 47%, with a weighted mean between 20% and 22% (Derks and Tomasi 2015; Mombelli et al. 2012). These discrepancies largely reflect differences in case definitions prior to the establishment of uniform criteria by the 2017 World Workshop of the American Academy of Periodontology (AAP) and the European Federation of Periodontology (EFP). Peri‐implantitis should no longer be considered a rare condition, as it may affect approximately one‐third of patients and one‐fifth of dental implants within a given population (Kordbacheh Changi et al. 2019).

The role of biofilm in the onset and progression of peri‐implant diseases has been extensively explored (Pontoriero et al. 1994; Zitzmann et al. 2001). While clinicians often draw parallels between periodontitis and peri‐implantitis lesions, both exhibit distinct histological differences (Carcuac et al. 2013; Carcuac and Berglundh 2014) and microbial environments (Robitaille et al. 2016). Peri‐implantitis lesions are generally larger, with higher proportions and densities of plasma cells (CD138+), macrophages (CD68+), and myeloperoxidase‐positive (MPO+) cells compared to periodontitis lesions (Carcuac and Berglundh 2014). Moreover, the inflammatory infiltrate in peri‐implantitis extends closer to the alveolar bone crest and demonstrates an increased rate of disease progression (Carcuac et al. 2013). Key anatomical aspects of the supracrestal tissue height and host–bacterial interactions between natural teeth and dental implants may explain differences in their pathogenesis and responses to oral dysbiosis.

Several known periodontal pathogens have been identified in peri‐implantitis. A recent systematic review identified *Staphylococcus epidermidis*, *Fusobacterium nucleatum*, *Treponema denticola*, *Tannerella forsythia*, *Prevotella intermedia*, and *Porphyromonas gingivalis* to be associated with biofilm from peri‐implantitis lesions (Carvalho et al. 2023). Moreover, multiplexed bacterial tag‐encoded FLX amplicon pyrosequencing confirmed that the peri‐implant microbiome demonstrated significantly less diversity than natural teeth and displayed distinct bacterial lineages in both health and disease among both ecosystems (Dabdoub et al. 2013). An observational study evaluating colonization dynamics of the pristine peri‐implant sulcus found that pioneer species serve as anchors for influential microbial hubs, facilitating biofilm maturation and recruitment of new bacterial community members (Dutra et al. 2025).

**Figure 1:**
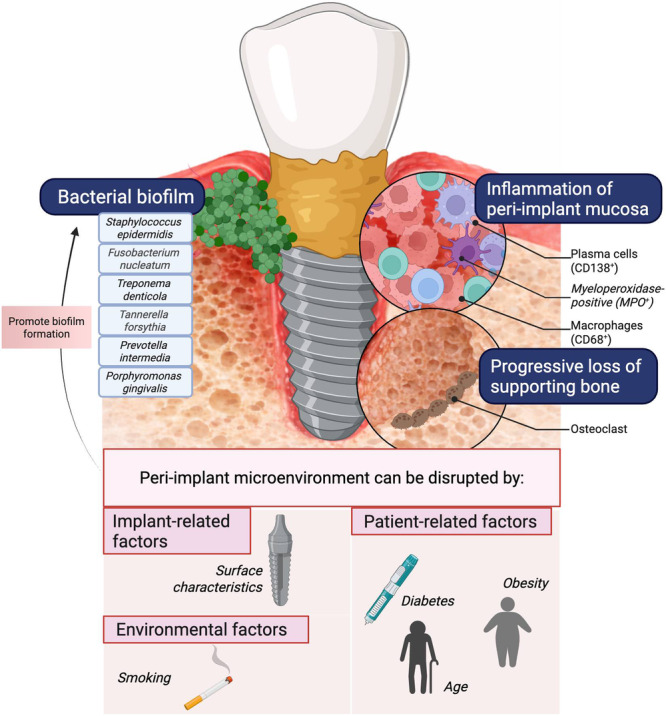
Peri‐implant microenvironment.

Despite its resilience, the peri‐implant microbiome can be disrupted by patient‐related factors (e.g., diabetes mellitus), environmental factors (e.g., smoking), and implant‐related factors (e.g., surface characteristics), as shown in Figure 1. These disruptions may promote the formation of biofilms distinct from those on natural teeth (Longo et al. 2018; Martínez‐Hernández et al. 2016; Sinjab et al. 2024; Tsigarida et al. 2015). Therefore, understanding host immune mechanisms in peri‐implantitis requires OMICS‐based approaches to capture microbial dynamics and their interactions with the host immune system.

OMICS technologies are high‐throughput approaches for large‐scale analysis of biological molecules, enabling the generation of extensive data sets and providing new insights into complex biological systems (Committee on the Review of Omics‐Based Tests for Predicting Patient Outcomes in Clinical Trials et al. 2012; Dai and Shen 2022). These disciplines include: genomics (large‐scale analysis of DNA sequence), epigenomics (large‐scale analysis of reversible chemical modifications to DNA and histones), transcriptomics (large‐scale analysis of coding and non‐coding RNAs, including messenger RNAs “mRNAs,” microRNAs “miRNAs,” long non‐coding RNAs “lncRNAs,” and circular RNAs “circRNAs”), proteomics (large‐scale analysis of proteins), metabolomics (large‐scale analysis of small‐molecule metabolites), and microbiomics (large‐scale characterization of microbial communities within a given environment) (Committee on the Review of Omics‐Based Tests for Predicting Patient Outcomes in Clinical Trials et al. 2012; Dai and Shen 2022).

Furthermore, OMICS technologies have transformed our ability to investigate complex biological systems. By generating multidimensional data sets and applying advanced bioinformatics, these approaches enable comprehensive study of molecular features across multiple biological layers, facilitating a better understanding of host immune responses, genetic predisposition, environmental influences, and their contributions to disease (Hasin et al. 2017; Karczewski and Snyder 2018), with specific relevance to peri‐implantitis.

Thus, the purpose of the present review is to evaluate current and emerging OMICS technologies used to study peri‐implantitis from a host immune perspective, and their contribution to the understanding of peri‐implantitis pathogenesis. Microbiomics studies are discussed elsewhere.

## 2 Search Strategy and Literature Selection

This narrative review was conducted to provide a comprehensive overview of OMICS technologies and their contributions to understanding peri‐implantitis pathogenesis. Electronic and manual searches in PubMed, MEDLINE and Google Scholar databases were performed up to October 2025 using search terms combining “peri‐implantitis” or “periimplantitis” with OMICS‐related terms, including “genomics,” “epigenomics,” “transcriptomics,” “proteomics,” “metabolomics,” “microbiome,” “metagenomics,” “RNA‐seq,” “single‐cell,” “spatial transcriptomics,” “multi‐omics,” and “machine learning.” No restrictions were placed on publication date. Additional relevant articles were identified through manual screening of reference lists of retrieved articles and the authors' expertise in periodontal and peri‐implant research.

Article selection (e.g., randomized clinical trials, prospective/retrospective cohorts, and case series) was based on clinical relevance and scientific contribution to the understanding of peri‐implantitis pathogenesis from a host immune perspective, without application of formal inclusion/exclusion criteria or standardized quality assessment tools. Background and contextual literature on OMICS methodologies, peri‐implant biology, and related foundational concepts were also incorporated to provide comprehensive coverage of the topic and facilitate the reader's understanding of the technological approaches discussed.

## 3 Role of OMICS Technologies in Elucidating the Pathophysiology of Peri‐Implantitis

### 3.1 Genomics

Genomics refers to the large‐scale analysis of the complete sequence of DNA in an organism (Committee on the Review of Omics‐Based Tests for Predicting Patient Outcomes in Clinical Trials et al. 2012). To date, only one study has analyzed the genomic landscape of peri‐implantitis using whole‐exome sequencing (WES), which targets protein‐coding regions (Lee et al. 2014). In this study, six unrelated, nonsmoking patients with clustered implant failures due to severe peri‐implantitis were enrolled. Saliva samples were collected from a total of 26 failed implants, and WES was performed to identify variants with high or moderate impact, including loss‐of‐function changes. Common variants (minor allele frequency ≥ 0.05) were excluded using reference data sets (dbSNP, 1000 Genomes East Asian population, and a healthy Korean subset from the GSK project), resulting in a final set of 2022 protein‐coding genes (Lee et al. 2014).

Functional analysis revealed multiple enriched gene sets associated with cytoskeletal organization, cell adhesion, and metal ion binding. Network analysis highlighted metal ion binding genes as central hubs connecting distinct functional clusters, suggesting that dysregulation of metal ion homeostasis may affect cell morphology, osteoblast and epithelial adhesion, and peri‐implant tissue integrity. Genes previously associated with peri‐implantitis and periodontitis were also specifically examined in the case cohort, and variants in interleukin 1 alpha (IL‐1A), interleukin 1 beta (IL‐1B), and tumor necrosis factor (TNF) were detected within the data set (Lee et al. 2014). These findings link host genomic variation to peri‐implant tissue dysfunction, which may contribute to implant failure (Lee et al. 2014).

Despite the limited sample size and absence of functional validation, this study suggests that host genomic variation may contribute to peri‐implant tissue susceptibility through genes involved in cytoskeletal organization, cell adhesion, metal ion homeostasis, and inflammatory signaling pathways. These findings implicate genetic determinants in maintaining peri‐implant tissue integrity and provide preliminary insight into host factors that may predispose individuals to severe peri‐implantitis.

### 3.2 Epigenomics

Epigenomics is the large‐scale study of the epigenome, which includes reversible chemical modifications of DNA and histone proteins that modulate gene expression without altering the underlying DNA sequence (Committee on the Review of Omics‐Based Tests for Predicting Patient Outcomes in Clinical Trials et al. 2012). To date, the first study integrating epigenomic and transcriptomic analyses in peri‐implantitis was conducted by Cho et al. (2020). In this study, gingival tissue samples were collected from 20 patients, divided into four groups: peri‐implantitis nonsmokers, peri‐implantitis smokers, periodontitis nonsmokers, and periodontitis smokers, with five patients per group. The methylome was assessed using reduced representation bisulfite sequencing (RRBS), while transcriptome‐wide gene expression was analyzed using next‐generation RNA sequencing (RNA‐seq).

The study revealed that peri‐implantitis and periodontitis share some molecular features but also exhibit distinct patterns of gene expression and DNA methylation. Smoking was found to modulate these patterns differently in the two disease types. In periodontitis, smoking‐associated methylation changes were primarily linked to extracellular matrix (ECM) organization, suggesting that smoking may accelerate disease progression through structural tissue alterations. In peri‐implantitis, however, smoking predominantly affected genes related to cell division, indicating that peri‐implant tissues are more sensitive to environmental risk factors. Across all groups, DNA methylation was negatively correlated with gene expression, consistent with canonical epigenetic regulation, and regression analyses suggested a higher epigenetic responsiveness to smoking in peri‐implantitis compared with periodontitis (Cho et al. 2020).

Importantly, the directionality of methylation–expression coupling was illustrated by representative differentially methylated (DM) genes. In peri‐implantitis (nonsmokers vs. smokers), genes such as solute carrier family 1 member 5 (SLC1A5) and tenascin C (TNC) demonstrated increased DNA methylation in smokers, accompanied by reduced gene expression, reflecting a classical inverse relationship between DNA methylation changes and gene expression. Similar inverse‐direction patterns were observed for additional highlighted genes, including G protein‐coupled receptor class C group 5 member C (GPRC5C) and RAD54‐like (RAD54L). In periodontitis, smoking‐associated changes also followed an inverse methylation–expression trend in representative genes, although these were functionally enriched in ECM‐related pathways rather than cell division. These findings clarify that the primary difference between diseases lies not in the direction of regulation, predominantly inverse in both cases, but in the biological pathways most affected by smoking.

Functional and network analyses identified several genes and pathways potentially contributing to peri‐implant disease mechanisms. Genes involved in immune regulation, including interleukin 17 receptor C (IL17RC), C‐C motif chemokine ligand 19 (CCL19), and type I interferon‐related genes, were associated with neutrophil behavior and host defense responses. Glutamate ionotropic receptor NMDA type subunit 2 A (GRIN2A) was linked to bone metabolism, leiomodin 1 (LMOD1) to cytoskeletal dynamics, and SLC1A5 to defense against foreign body formation. Zinc finger proteins (ZNF354C, ZNF468) and TNC were implicated in transcriptional regulation, cell signaling, and inflammatory responses. Gene ontology enrichment further highlighted ciliary function, defense response, and immune activation as key features distinguishing peri‐implantitis from periodontitis, while network analyses suggested that smoking may indirectly promote inflammation and tissue disruption through these molecular pathways (Cho et al. 2020). Nevertheless, gene‐level methylation changes should be interpreted with caution, as the study included only five patients per subgroup, and used RRBS, which provides limited coverage of the methylome. 

Despite the small sample size and the limited evaluation of additional epigenetic mechanisms, this study provides evidence that differential DNA methylation patterns contribute to the distinct molecular landscape of peri‐implantitis compared with periodontitis and may modulate host responses to environmental exposures such as smoking (Cho et al. 2020). Notably, DNA methylation was negatively correlated with gene expression across key immune, signaling, and cell‐regulatory genes, linking epigenetic changes directly to functional alterations in host responses. These findings support the role of epigenetic regulation in shaping immune and inflammatory pathways in peri‐implantitis and warrant further investigation of methylation‐driven mechanisms in disease susceptibility and progression.

### 3.3 Transcriptomics

Transcriptomics is the study of the complete set of RNA transcripts, including mRNAs and non‐coding RNAs, such as miRNAs, lncRNAs, and circRNAs, under specific physiological or pathological conditions (Committee on the Review of Omics‐Based Tests for Predicting Patient Outcomes in Clinical Trials et al. 2012). Profiling gene expression at the tissue or single‐cell level provides a dynamic view of cellular responses and molecular pathways, offering mechanistic insights into host immune responses, inflammation, tissue remodeling, and bone metabolism in peri‐implantitis. High‐throughput RNA‐seq technologies, including bulk RNA‐seq, single‐cell RNA sequencing (scRNA‐seq), and emerging spatial transcriptomics, enable comprehensive characterization of gene expression landscapes, as well as distinct cellular and molecular signatures (Margulies et al. 2005; Marx 2021; Ståhl et al. 2016; F. Tang et al. 2009).

Building upon these transcriptomic approaches, Ganesan et al. explored the interplay between the peri‐implant microbiome and host gene expression by analyzing paired plaque and gingival samples from healthy and diseased implants, collected from five nonsmoking patients (Ganesan et al. 2022). In diseased sites, microbial genes related to biofilm thickness, heme transport and utilization, and gram‐negative cell membrane synthesis were upregulated 4‐ to 200‐fold. These microbial changes correlated with host gene expression patterns involving apoptosis, mucosal zinc finger proteins (ZNFs), membrane transport, inflammation, and cell‐cell communication. Notably, 112 out of 632 ZNFs, which are transcription factors related to DNA methylation and nuclear factor kappa‐B (NFκB) signaling, were significantly upregulated in peri‐implantitis and showed strong positive correlations with bacterial virulence factors. Despite the small sample size, these findings highlight key host–microbial interactions and provide a foundation for future mechanistic studies and targeted interventions.

Complementing this host‐microbiome perspective, Martin et al. provided a tissue‐level view of peri‐implantitis through transcriptome‐wide profiling of gingival tissue biopsies from seven healthy nonsmoking patients, including three with peri‐implant health and four with peri‐implantitis (Martin et al. 2022). Using a next‐generation microarray covering over 20,000 genes, the study revealed significant upregulation of genes associated with intracellular trafficking and stress responses in diseased tissues. Specifically, WASH complex subunit 1 (WASH1), involved in phagosome maturation, and biogenesis of lysosomal organelles complex 1 subunit 4 (BLOC1S4), involved in lysosome biogenesis, were linked to the endosomal‐lysosomal pathway, suggesting increased intracellular processing in response to bacteria and titanium particles. DnaJ heat shock protein family (Hsp40) member C28 (DNAJC28), a heat shock response gene, and cyclin‐dependent kinase 12 (CDK12), a regulator of transcriptional maintenance genes, were also upregulated, indicating enhanced cellular stress and modulation of gene expression programs in peri‐implantitis. Cellular respiration pathways involved in oxidative stress were highly transcribed across all peri‐implant samples, implying that implant‐specific factors maintain a persistent oxidative microenvironment. While limited by small sample size and lack of functional validation, this study establishes a foundation for understanding intracellular and stress‐related mechanisms in peri‐implantitis.

Based on the previously mentioned observations, subsequent studies have applied bulk RNA‐seq combined with machine learning to better understand the immune environment in peri‐implantitis. One study demonstrated that the peri‐implant immune milieu reflected clinical risk, shaped microbial composition, and influenced regenerative treatment outcomes (Wang et al. 2021). By classifying granulation tissue from diseased implants into low‐, intermediate‐, and high‐risk groups based on immune cell composition, the authors correlated immune profiles with clinical outcomes. The low‐risk group exhibited higher M1/M2 macrophage ratios, reduced B cells, increased CD4^+^ and Treg cells, enhanced complement signaling, and elevated Th1 and Th17 cytokine levels (Wang et al. 2021). Similarly, weighted gene co‐expression network analysis highlighted upregulated genes including immunoglobulin lambda like polypeptide 5 (IGLL5), signal sequence receptor subunit 4 (SSR4), marginal zone B and B1 cell specific protein (MZB1), and X‐box binding protein 1 (XBP1), implicating Interleukin‐2/Signal Transducer and Activator of Transcription 5 (IL‐2/STAT5)‐mediated activation of immune cells as a key mechanism in peri‐implantitis (L. Tang et al. 2022). These findings highlight the importance of immune cell composition and signaling networks in disease progression.

To address the limitations of bulk analyses, single‐cell RNA sequencing (scRNA‐seq) has been applied to characterize cellular heterogeneity within peri‐implantitis lesions. Mo et al. reported an increased prevalence of CXCR2+ neutrophils and a pro‐inflammatory fibroblast subpopulation characterized by elevated expression of the following C‐X‐C motif chemokine ligands (CXCL): CXCL1, CXCL2, CXCL6, CXCL8, CXCL13, and IL‐24 (Mo et al. 2024). Enhanced CXCL8+ fibroblast–CXCR2+ neutrophil interactions were identified as a critical mechanism driving sustained inflammation. In a complementary study, Li et al. observed reduced stromal cell numbers and increased immune cells in peri‐implantitis tissues (Li et al. 2024). A fibroblast subpopulation expressing CXCL13 recruited neutrophils through specific chemokine‐receptor interactions, while monocytes and macrophages were more likely to differentiate into osteoclasts, providing a mechanistic explanation for the rapid marginal bone loss characteristic of peri‐implantitis.

More recently, spatial transcriptomics has been applied by Dionigi et al. (2026) to preserve tissue architecture while evaluating gene expression in peri‐implantitis. Soft tissue biopsies from peri‐implantitis and reference implant sites were analyzed using spatial transcriptomics (*n* = 4), RNA‐sequencing (*n* = 20), and immunohistochemistry (*n* = 38). Peri‐implantitis sites showed higher overall gene activity and clear associations between distinct gene clusters and specific tissue compartments. Gene‐set enrichment analysis revealed dysregulation of pathways related to antimicrobial host response, collagen fibril organization, and angiogenesis. Immunohistochemistry confirmed elevated protein levels corresponding to the most upregulated genes, providing spatially resolved validation of transcriptional alterations. This study highlights the added value of spatial resolution in understanding tissue‐specific gene expression patterns in peri‐implantitis.

Finally, oral fluid specimens, such as peri‐implant crevicular fluid (PICF), provide a noninvasive alternative for transcriptomic biomarker analysis. In a prospective study (Menini et al. 2021), seven patients with fixed partial dentures supported by 14 implants were followed over 5 years, and PICF and peri‐implant mucosal tissue were collected 3 months post‐implant insertion. Microarray analysis of miRNAs revealed that specific extracellular miRNA profiles in PICF were predictive of subsequent marginal bone resorption, with 14 miRNAs—miR‐34a, miR‐100, miR‐106a, miR‐126, miR‐143, miR‐146a, miR‐181, miR‐200, miR‐221, miR‐223, miR‐375, miR‐378, miR‐429, and miR‐1248—showing altered expression both in PICF and in the corresponding soft tissue at sites with bone resorption > 1 mm. No implant failures occurred during follow‐up, and the mean bone loss was 1.98 mm. These findings demonstrate that miRNAs in PICF can serve as site‐specific, noninvasive biomarkers, effectively reflecting tissue‐level changes and providing a liquid biopsy approach for monitoring peri‐implant bone resorption. This approach offers translational potential for patient‐specific risk assessment, early diagnosis, and intervention.

Despite the relatively small sample sizes and limited functional validation in current studies, transcriptomic analyses provide compelling evidence that peri‐implantitis is characterized by dynamic alterations in host gene expression. Bulk, single‐cell, and spatial transcriptomics collectively reveal dysregulated immune signaling, pro‐inflammatory fibroblast–neutrophil interactions, enhanced stress‐response pathways, and impaired stromal–immune homeostasis. Furthermore, extracellular RNA signatures, particularly miRNAs in PICF, reflect tissue‐level changes and offer noninvasive biomarkers for site‐specific disease monitoring. These findings emphasize the central role of transcriptional regulation in mediating host responses to microbial and environmental cues, and provide a foundation for mechanistic studies, predictive biomarker development, and targeted therapeutic interventions in peri‐implantitis.

### 3.4 Proteomics

Proteomics is the study of the complete set of proteins expressed by a cell, tissue, or organism (Committee on the Review of Omics‐Based Tests for Predicting Patient Outcomes in Clinical Trials et al. 2012). Existing studies of peri‐implantitis using proteomics have primarily focused on the protein composition of PICF. The first proteomics study to analyze PICF applied liquid chromatography‐tandem mass spectrometry (LC‐MS/MS) to samples from 25 patients before and after regenerative treatment of peri‐implantitis‐affected implants (Esberg et al. 2019). The aim was to identify associations between PICF proteomic profiles and treatment outcomes, including implant failure or survival, bleeding on probing (BOP), pocket depth (PD), and the use of enamel matrix derivative (EMD). Principal component analysis of longitudinal PICF proteomic profiles revealed distinct clusters of patients: one cluster associated with active peri‐implantitis and subsequent implant loss, and another with implant survival and positive outcomes following EMD treatment. These findings highlight that specific PICF proteomic signatures reflect the activity of peri‐implantitis and may serve as potential prognostic indicators.

Halstenbach et al. conducted a pilot proteomic study comparing 38 PICF samples from healthy implants, diseased implants, and healthy teeth across 14 patients (Halstenbach et al. 2023). A total of 2332 human proteins were identified, and while no differences were observed between healthy teeth and healthy implants, peri‐implantitis samples exhibited substantial deviations. Specifically, 59 proteins were significantly upregulated and 31 downregulated in peri‐implantitis. Upregulated proteins included neutrophil‐expressed antimicrobial proteins, such as myeloperoxidase (MPO), bactericidal/permeability‐increasing protein (BPI), azurocidin, and cathelicidin, alongside inflammatory proteins including immunoglobulin heavy variable 2‐D, leukotriene A‐4 hydrolase (LTA4H), dysferlin, and speckled protein 100 (SP100). Gene ontology analysis further revealed elevated immunological and proteolytic activity in peri‐implantitis samples, reflecting neutrophil‐dominated inflammation. Additionally, 334 bacterial proteins were identified, suggesting that microbial activity contributes to the inflammatory environment observed in diseased implants.

Collectively, both studies highlight that peri‐implantitis is characterized by dysregulated immune and proteolytic activity, predominantly driven by neutrophil‐derived antimicrobial proteins and inflammatory mediators. Disease‐specific protein signatures correlate with clinical outcomes, including tissue inflammation and implant survival, and highlight the interplay between host immune responses and microbial activity. However, the limited number of studies and small sample sizes necessitate further validation.

### 3.5 Metabolomics

Metabolomics is the study of the complete set of small molecule metabolites within a biological sample (Committee on the Review of Omics‐Based Tests for Predicting Patient Outcomes in Clinical Trials et al. 2012). While considerable research has explored the microbiome, transcriptome, and proteome of peri‐implantitis, characterization of the metabolic landscape remains limited. Previous transcriptomic studies suggested that in health, most microbial transcripts and over 50% of the mucosal transcriptome relate to metabolic processes, indicating an active crosstalk between the peri‐implant microbiome and host tissue that maintains barrier function (Ganesan et al. 2022). However, direct evidence of this metabolic interaction on a global level, and its implications for peri‐implantitis pathogenesis remains scarce.

Mass spectrometry (MS)‐based approaches have enabled both targeted and non‐targeted analyses of PICF metabolites in health and peri‐implantitis. A non‐targeted metabolomic study of 56 patients revealed 179 distinct metabolites, with 169 significantly elevated in peri‐implantitis compared with healthy implants (Song et al. 2024). Among these, amino acids, fatty acids, and nucleotides were most differentially abundant. Targeted analysis further highlighted higher levels of organic acids, including fructose‐6‐phosphate, glucose‐6‐phosphate, and succinic acid, in peri‐implantitis. Succinic acid correlated positively with the abundance of pathogenic genera such as *Porphyromonas* and *Treponema*, and negatively with *Streptococcus*. These findings suggest a microbiome‐metabolome crosstalk that may contribute to the metabolic dysregulation observed in peri‐implantitis and mirror patterns reported in periodontitis (Song et al. 2024).

Complementing these observations, Alassy et al. used 1H‐NMR spectroscopy to analyze 126 implants across health, mucositis, and peri‐implantitis conditions (Alassy et al. 2025). Among the 35 metabolites identified, cadaverine, lysine, and putrescine were strongly associated with peri‐implantitis, whereas alpha‐ketoglutarate correlated with implant health. Moreover, proline and 1‐3‐diaminopropane levels predicted future bone loss, while biotin, propionate, and valine predicted radiographic bone level (RBL) stability over time. These results demonstrate that specific metabolic signatures in PICF not only distinguish peri‐implantitis from health but may also serve as predictive markers for future peri‐implant bone loss and disease progression.

Taken together, these studies indicate that peri‐implantitis is characterized by distinct metabolic alterations in PICF, reflecting both host tissue responses and microbial contributions. Disease‐specific metabolite profiles correlate with progression of bone loss and tissue inflammation, highlighting the potential of metabolic signatures as diagnostic and prognostic biomarkers. However, due to a limited number of studies, small sample sizes, and challenges in establishing causal links between metabolites and disease mechanisms, these findings require confirmation in larger, independent cohorts.

## 4 Integrative Synthesis of OMICS Findings in Peri‐Implantitis

### 4.1 Immune Regulation

Integration across OMICS layers highlights immune dysregulation as a central feature of peri‐implantitis. Genomic analyses indicate that variants in genes involved in metal ion binding, cytoskeletal organization, and cell adhesion may prime peri‐implant tissues for altered immune and structural responses, potentially sensitizing them to bacterial insults and environmental stressors (Lee et al. 2014). Epigenomic analyses have identified gene‐specific DNA methylation changes, including IL17RC, CCL19, and type I interferon‐related genes, suggesting further epigenetic modulation of inflammatory pathways (Cho et al. 2020). ZNFs, such as ZNF354C and ZNF468, are proposed to act as transcriptional regulators linking epigenetic modifications to NFκB‐mediated immune responses (Cho et al. 2020).

Transcriptomic studies complement these findings, demonstrating upregulation of cytokines, chemokines, and cell‐type‐specific immune interactions, including CXCL8+ fibroblast–CXCR2+ neutrophil crosstalk and fibroblast‐mediated neutrophil recruitment, which sustain localized inflammation and enable rapid immune cell mobilization (Ganesan et al. 2022; Li et al. 2024; Martin et al. 2022; Mo et al. 2024; L. Tang et al. 2022; Wang et al. 2021). Proteomic profiling of PICF further confirms translation of transcriptional programs into protein‐level responses, with elevated neutrophil‐derived antimicrobial proteins (e.g., MPO, BPI, azurocidin, and cathelicidin) and inflammatory mediators (e.g., LTA4H, SP100, dysferlin, and immunoglobulin heavy variable 2‐D) reflecting active host defense (Esberg et al. 2019; Halstenbach et al. 2023). Metabolomic analyses show accumulation of succinic acid, amino acids, cadaverine, lysine, and putrescine in peri‐implantitis, which correlate with pathogenic microbial genera such as *Porphyromonas* and *Treponema*, and inversely with commensals like *Streptococcus*, illustrating metabolite‐mediated immune modulation and host–microbiome crosstalk (Alassy et al. 2025; Song et al. 2024). Collectively, these data reveal convergent immune pathways driven by host genetic priming, epigenetic modifications, transcriptional orchestration, translation into functional protein responses, and metabolic regulation.

### 4.2 Tissue Remodeling and ECM Organization

OMICS layers consistently highlight dysregulated tissue remodeling as a hallmark of peri‐implantitis. Transcriptomic analyses demonstrate upregulation of genes involved in extracellular ECM organization, oxidative stress responses, and epithelial–mesenchymal transition (EMT), suggesting enhanced tissue turnover and structural disruption (Martin et al. 2022; L. Tang et al. 2022; Wang et al. 2021). Proteomic studies support these observations, revealing alterations in matrix‐degrading enzymes and structural proteins in PICF, including neutrophil‐associated proteases and inflammatory mediators (Esberg et al. 2019; Halstenbach et al. 2023). Metabolomic signatures, including elevated amino acids and organic acids, may further modulate fibroblast activity and ECM remodeling, linking metabolic shifts to tissue‐level changes (Alassy et al. 2025; Song et al. 2024). Genomic variants affecting adhesion, cytoskeleton, and ECM‐interacting proteins may predispose tissues to heightened sensitivity to these dysregulated remodeling pathways, providing a priming layer that interacts with epigenetic, transcriptional, and metabolic mechanisms (Lee et al. 2014). Taken together, these findings suggest that tissue breakdown in peri‐implantitis is orchestrated through multilayered molecular events.

### 4.3 Host–Microbiome Interactions

Multi‐OMICS data highlight the complex interactions between microbial activity and host responses. Transcriptomic studies, including bulk, single‐cell, and spatial approaches, reveal that immune signaling is spatially organized to respond to microbial stimuli, with specific fibroblast and neutrophil subpopulations coordinating local defense (Dionigi et al. 2026; Ganesan et al. 2022; Li et al. 2024; Martin et al. 2022; Mo et al. 2024; L. Tang et al. 2022; Wang et al. 2021). Metabolomic analyses demonstrate that microbial metabolism shapes the chemical microenvironment: specific organic acids, especially succinic acid, correlate positively with pathogenic taxa and negatively with commensals, reinforcing feedback loops between microbial activity and host immune responses (Song et al. 2024). Proteomic findings confirm neutrophil recruitment and antimicrobial protein enrichment at microbial challenge sites, reflecting the translation of transcriptomic signals into functional immune responses (Esberg et al. 2019; Halstenbach et al. 2023). Genomic variants may further influence host susceptibility and responsiveness to microbial cues, priming tissues for amplified inflammatory and tissue‐destructive responses (Lee et al. 2014). This integrated view highlights how host–microbe interactions drive chronic inflammation and tissue damage in peri‐implantitis.

### 4.4 Integrated Perspective of Peri‐Implantitis Pathogenesis

Combining observations from genomics, epigenomics, transcriptomics, proteomics, and metabolomics reflect that peri‐implantitis is as a multilayered host–microbiome molecular ecosystem. Genomic variants set the stage by priming host tissues for heightened sensitivity to environmental and microbial challenges. Epigenetic modifications, including ZNFs, may further contribute to cellular responsiveness. Transcriptional signals coordinate cytokine and chemokine networks, which are executed at the protein level and amplified or modulated by metabolic shifts that reflect microbial activity. Taken together, these processes drive chronic immune dysregulation, ECM disruption, tissue remodeling, and progressive bone loss.

**Figure 2:**
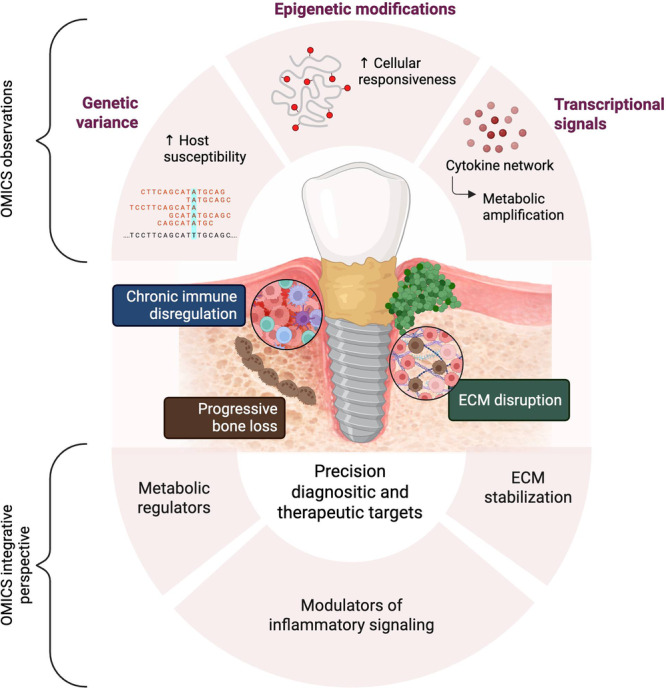
Integrated host–microbiome molecular ecosystem driving disease pathogenesis.

This integrative perspective identifies convergent molecular pathways suitable for diagnostics, prognostics, and precision therapies, including modulators of inflammatory signaling, ECM stabilization, and metabolic regulators. Multi‐OMICS analyses characterize the mechanistic basis of peri‐implantitis pathogenesis (Alassy et al. 2025; Cho et al. 2020; Dionigi et al. 2026; Esberg et al. 2019; Ganesan et al. 2022; Halstenbach et al. 2023; Lee et al. 2014; Li et al. 2024; Martin et al. 2022; Mo et al. 2024; Song et al. 2024; L. Tang et al. 2022; Wang et al. 2021). These processes are summarized schematically in Figure 2, which illustrates the integrated host–microbiome molecular ecosystem driving disease pathogenesis.

## 5 Future Directions, Translational Potential, and Limitations

### 5.1 Future Directions

Integration of OMICS layers—including genomics, epigenomics, transcriptomics, proteomics, metabolomics, and microbiomics—offers a pathway toward precision peri‐implant care. Moving from single‐platform studies to vertically integrated, patient‐centered data sets will allow mechanistic cascades to be mapped and modeled, enabling predictive analyses for individual implant sites. Computational modeling approaches may simulate tissue responses to microbial shifts, implant surface characteristics, or host‐modulation therapies (Samei 2025), providing a framework for future development of multi‐analyte biomarker panels.

### 5.2 Translational Potential

Multi‐OMIC biomarker panels integrating microbial taxa, host proteins, and metabolites from sub‐microlitre PICF or saliva have demonstrated improved diagnostic performance relative to conventional probing, detecting disease at a stage when bone loss is still reversible (Condor et al. 2025; Fan et al. 2023; Pais et al. 2025). Computational modeling may also help identify key pathogenic targets for tailored interventions, including phage therapies or probiotics, and support precision modulation of host responses through programmable epigenetic approaches. Integrating OMICS data into predictive models, rapid diagnostics, and site‐specific therapies may improve diagnostic accuracy and treatment outcomes of peri‐implantitis.

### 5.3 Limitations and Challenges

Despite their potential, there are several limitations and challenges that need to be addressed in OMICS research in peri‐implantitis:

- *Sample limitations*: Restricted tissue and fluid volumes, heterogeneous cell populations, and diverse sample types (soft tissue, PICF, and saliva) complicate molecular analyses and limit direct comparability across studies.
- *Cohort limitations*: Small, heterogeneous patient cohorts with variable case definitions and ethnic diversity reduce reproducibility.
- *Molecular and analytical challenges*: RNA degradation, extreme protein and metabolite dynamic ranges, low‐abundance transcript detection, and high‐dimensional but sample‐limited data sets complicate cross‐study comparison and independent validation.
- *Environmental and biological variability*: Diet, circadian rhythms, oral hygiene, and smoking may confound metabolomic and epigenetic measurements.
- *Spatial complexity*: Sectioning titanium‐bearing tissues without molecular delocalization remains technically challenging; spatial concentration gradients are often inferred rather than directly measured.
- *Data harmonization and validation*: Lack of standardized metadata and open‐access repositories restricts cross‐study comparison; predictive multi‐OMICS classifiers frequently underperform in independent cohorts.

Addressing these limitations will require carefully designed cohorts, standardized sampling protocols, and robust computational frameworks to fully realize OMICS‐driven understanding and management of peri‐implantitis (Hasin et al. 2017; Karczewski and Snyder 2018).

## 6 Conclusions

Advances in OMICS technologies have deepened our understanding of peri‐implantitis, revealing it as a complex host–microbiome molecular ecosystem. Integration of genomics, epigenomics, transcriptomics, proteomics, and metabolomics shows coordinated molecular networks driving immune dysregulation, ECM disruption, tissue remodeling, and metabolic changes.

Host genetic variation shapes susceptibility and downstream molecular responses, while epigenetic alterations, including zinc finger protein–associated regulation, modulate immune‐related gene expression. These transcriptional changes align with proteomic profiles of neutrophil recruitment and inflammation, and metabolomic shifts that both reflect and sustain tissue damage. Taken together, these findings demonstrate that peri‐implantitis progression involves interconnected molecular processes across multiple layers, with site‐specific and spatially organized host responses.

These insights lay the groundwork for the development of biomarker panels, predictive models, and targeted host‐modulatory or antimicrobial therapeutic strategies. Future studies should prioritize integrated, single‐cell, and spatially resolved OMICS approaches with longitudinal sampling to further characterize the mechanisms underlying peri‐implantitis pathogenesis and translate findings into precise clinical interventions.

## Author Contributions

F.A. conceived the main idea. F.A., A.H., K.Y., P.M.G.‐O., and C.G.‐P. contributed to the design, writing, and critical review of the manuscript. R.L.‐J. contributed to the writing of the manuscript. A.C. contributed to the critical revision of the manuscript. P.M.G.‐O. contributed to the creation of figures. All authors have read and agreed to the final version of the manuscript.

## Conflicts of Interest

The authors declare no conflicts of interest.

## Data Availability

Data sharing does not apply to this article as no new data were created or analyzed in this study.
